# Public and private maternal health service capacity and patient flows in southern Tanzania: using a geographic information system to link hospital and national census data

**DOI:** 10.3402/gha.v7.22883

**Published:** 2014-01-09

**Authors:** Patrik Tabatabai, Stefanie Henke, Katharina Sušac, Oberlin M. E. Kisanga, Inge Baumgarten, Gisela Kynast-Wolf, Heribert Ramroth, Michael Marx

**Affiliations:** 1 Institute of Public Health, University of Heidelberg, Heidelberg, Germany; 2 Laboratory for Geodata Analysis and Visualization, Department III Civil Engineering and Geoinformation, Beuth University, Berlin, Germany; 3 Tanzanian German Programme to Support Health, German Development Cooperation/Deutsche Gesellschaft fuer Internationale Zusammenarbeit GmbH, Dar es Salaam, Tanzania; 4 Epidemiology and Biostatistics, Institute of Public Health, University of Heidelberg, Heidelberg, Germany

**Keywords:** public private, maternal health, Tanzania, faith-based, service capacity, health mapping

## Abstract

**Background:**

Strategies to improve maternal health in low-income countries are increasingly embracing partnership approaches between public and private stakeholders in health. In Tanzania, such partnerships are a declared policy goal. However, implementation remains challenging as unfamiliarity between partners and insufficient recognition of private health providers prevail. This hinders cooperation and reflects the need to improve the evidence base of private sector contribution.

**Objective:**

To map and analyse the capacities of public and private hospitals to provide maternal health care in southern Tanzania and the population reached with these services.

**Design:**

A hospital questionnaire was applied in all 16 hospitals (public *n*=10; private faith-based *n*=6) in 12 districts of southern Tanzania. Areas of inquiry included selected maternal health service indicators (human resources, maternity/delivery beds), provider-fees for obstetric services and patient turnover (antenatal care, births). Spatial information was linked to the 2002 Population Census dataset and a geographic information system to map patient flows and socio-geographic characteristics of service recipients.

**Results:**

The contribution of faith-based organizations (FBOs) to hospital maternal health services is substantial. FBO hospitals are primarily located in rural areas and their patient composition places a higher emphasis on rural populations. Also, maternal health service capacity was more favourable in FBO hospitals. We approximated that 19.9% of deliveries in the study area were performed in hospitals and that the proportion of c-sections was 2.7%. Mapping of patient flows demonstrated that women often travelled far to seek hospital care and where catchment areas of public and FBO hospitals overlap.

**Conclusions:**

We conclude that the important contribution of FBOs to maternal health services and capacity as well as their emphasis on serving rural populations makes them promising partners in health programming. Inclusive partnerships could increase integration of FBOs into the public health care system and improve coordination and use of scarce resources.

## Introduction

Little time is left to achieve the maternal health specific Millennium Development Goal 5. Yet, the annual death of around 290,000 women worldwide demonstrates that an efficient prevention of pregnancy-related deaths still presents a challenge (1). The presence of skilled health professionals at delivery improves delivery services, as well as perinatal care, and is strongly associated with the reduction of maternal mortality (2, 3). Despite the target of increasing skilled attendance at birth to 90% in 2015, the actual global percentage in 2008 was only 65.7%, with some countries having less than 20% coverage (4).

Strategies to overcome such unmet maternal health needs are increasingly embracing collaborative approachesbetween public and private (for-profit and not-for-profit) entities in health (5, 6). Among private not-for-profit stakeholders, faith-based organizations (FBOs) have a long-standing tradition as health service providers in low- and middle-income countries, accounting for 30–70% of the African health infrastructure (7). However, the establishment of stronger partnerships continues to be hampered by lacking recognition of FBOs’ contribution to health service delivery (7–9). In this context, the World Health Organization (WHO) called for greater dialogue between FBO and public health leaders, which should include the extension of health mappings to further specify FBOs’ role in health service provision (7). Yet, since that call by WHO in 2007, little has happened. In 2011, a systematic review examined the contribution of FBOs to maternal and newborn health in Africa over the past 20 years and found that only six articles were published on the subject (8). This notable gap in the academic literature was also acknowledged by Vogel et al. (2012), who stressed the need for greater research efforts in this field (9).

### The Tanzanian context

Tanzania is confronted with a high maternal mortality ratio of 454 per 100,000 live births, while having only 50.5% of births attended by skilled personnel. The service gap between rural and urban areas is remarkable with urban areas having nearly double the percentage of skilled births attendance than rural parts of the mainland (83.3% vs. 42.3%, respectively) (10). In an effort to increase accessibility to health care, the government of Tanzania introduced user-fee exemptions for maternal and child health services in all public health facilities (11, 12). However, extension of these exemption policies beyond the public health sector has been largely sporadic, and average costs for delivery increase substantially if FBO facilities are taken into account (13). The latter represent 40% of the country's hospitals, 22% of health centres and 13% of dispensaries (14). Partnering with the private health sector and in particular with FBOs is a declared policy goal (11), which has been adopted by Tanzania's *Strategy to Accelerate the Reduction of Maternal, Newborn and Child Death* (15). The approach seeks to improve access to quality health services by joint planning and a more rational use of resources, while cutting back duplication and unhealthy competition. Efforts to increase collaboration include contracting of FBO hospitals by the government to step in as *Designated District Hospitals* in districts without public hospital infrastructure. This is complemented by the transfer of budgetary allocations via basket funding or the *Service Agreement*, a contractual arrangement between local government authorities and private health facilities (16). However, implementation on the ground remains challenging, resulting in the perception of parallel systems, rather than collaborating and complementary partners (17). Misperceptions and unfamiliarity hamper utilization of possible synergies (17–19) and reflect the need to improve the visibility and evidence base of private sector contribution to health care provision in the country. Therefore, our study explored and mapped selected features of public and FBO hospital maternal health service capacity in southern Tanzania, as well as the population reached with these services.

## Methods

The study was conducted in 12 districts of southern Tanzania and included the administrative boundaries of Lindi and Mtwara Regions as well as Tunduru District of Ruvuma Region. Selection of the study area was purposive as this part of the country is supported by the Tanzanian German Programme to Support Health, meaning that ready access could be gained to district and hospital authorities. A multi-method approach using district, hospital and national data sources was applied.

### District questionnaire

District medical officers and members of the Council Health Management Teams were interviewed during site visits. Areas of inquiry included the number of: 1) *health facilities in the study area by type and managing authority*, and 2) *core medical professionals [medical doctors (MDs), assistant medical officers (AMOs), clinical officers (COs) and nurses] employed in these facilities*. Interviews were not conducted in Mtwara Urban and Lindi Urban due to time constraints. Information about the number of health facilities in the two districts was forwarded by local government authorities after the field research.

### Hospital questionnaire

A hospital questionnaire evaluated selected maternal health service indicators in all 16 hospitals in the study area (public *n*=10; FBO *n*=6). Among public hospitals, two were classified as level-2/regional hospitals, whereas all others hospitals were classified as level-1/district hospitals (11, 16). Key informants (medical officers in charge/members of the hospital management teams) were interviewed during site visits. Areas of inquiry included the availability of: 1) *human resources*, 2) *maternity and delivery beds*, as well as 3) *official provider-fees for obstetric services*.

*Human resource information* covered maternal health specific cadres (obstetricians/gynaecologists and maternity nurses) and other related and skilled personnel. In Tanzania, nurses can be differentiated into enrolled and registered nurses, with the latter undergoing longer training and having a higher level of qualification (20, 21).Other related and skilled cadres included MDs in general and mid-level cadres such as AMOs and COs. These cadres may not be primarily assigned to the maternity ward but are capable of fulfilling key tasks. AMOs, for example, are licensed to perform c-sections independently (22). *Official provider-fees for obstetric services* as reported by the hospital respondents were converted from Tanzanian Shillings (TZS) into USD using the average exchange rate for the year reviewed (23). Hospital admission and delivery books were reviewed to determine patient turnover for antenatal care (ANC), as well as normal deliveries and c-sections (period reviewed: January–December 2008).

### Mapping of patient flows and socio-geographic characteristics of maternal health service recipients

Global positioning system (GPS) coordinates were documented for all 16 hospitals (public *n*=10; FBO *n*=6) in the study area using a Garmin Ltd. Dakota^®^ 10 TOPO GPS recording device. In addition to the quantitative patient data, spatial information (patient origin by village/town) was transcribed from the paper-based records. The resulting database was linked to the 2002 Population and Housing Census dataset provided by the National Bureau of Statistics (NBS) (24) and the geographic information system software ESRI-ArcGIS Version 10. NBS Census data included a coding system for administrative units (village/town, ward, district and region) and a classification of wards (rural, urban and mixed), and both were adopted. This enabled assignment of patients to their corresponding ward of origin and the mapping of corresponding patient flows. The NBS ward classification was used to describe socio-geographic characteristics of public and FBO maternal health service recipients. Hospitals were defined eligible for analysis of patient composition if >70% of recorded patient visits were allocable to their corresponding ward of origin. This criterion was not met by the c-section data from one FBO hospital in Masasi District. GPS coordinates of villages/towns were not accessible, which is why exact distance to the hospital could not be measured.

NBS population figures were adjusted to annual population growth since the 2002 Census and the period reviewed using World Bank Indicators (25). The estimated number of annual births and pregnant women in the study area was approximated on the basis of an average crude birth rate (CBR) of 38.1 per 1,000 population for Tanzania Mainland (10, 26). The proportion of hospital births by ward was calculated using the number of traced hospital births as the numerator and the anticipated number of births as the denominator.

### Data analysis

Data was double-entered into an MSAccess database for further analysis. Descriptive statistics included averages, ranges and proportions, as appropriate. Student's *t*-test and Chi-square test were applied as required. The level of significance was set at *p*<0.05 and 95% confidence interval (CI) was used throughout. Statistical analysis was performed using STATA11.

### Informed consent and ethical clearance

Key respondents confirmed consent via signature. Ethical clearance for this study was granted by the Tanzania National Institute for Medical Research Review Committee and the Ethical Review Board at the University of Heidelberg, Germany.

## Results

### Study area characteristics

The study area of Lindi and Mtwara Regions as well as Tunduru District of the Ruvuma Region is divided into 12 districts and 249 wards, respectively. The area is populated by an estimated 2.5 million people, who primarily (70.5%) reside in rural areas (Table 1).

**Table 1:** Characteristics of the study area

	Rural		Urban		Mixed		Total
			
	*n*	%	*n*	%	*n*	%	*n*
Districts	10	83.3	2	16.7	0	0.0	12
Wards	190	76.3	21	8.4	38	15.3	249
Population	1,790,648	70.5	145,472	5.7	604,704	23.8	2,540,825
Men	854,459	70.4	70,138	5.8	288,390	23.8	1,212,986
Women	936,190	70.5	75,334	5.7	316,315	23.8	1,327,838
WCA	445,455	68.8	43,257	6.7	158,639	24.5	647,350

Rural–urban divide according to ward classification in the 2002 Census. Population figures are anticipated values for 2008 after adjusting for annual population growth since the 2002 Census (24, 25). WCA=women of childbearing age (15–49 years).

**Fig. 1:**
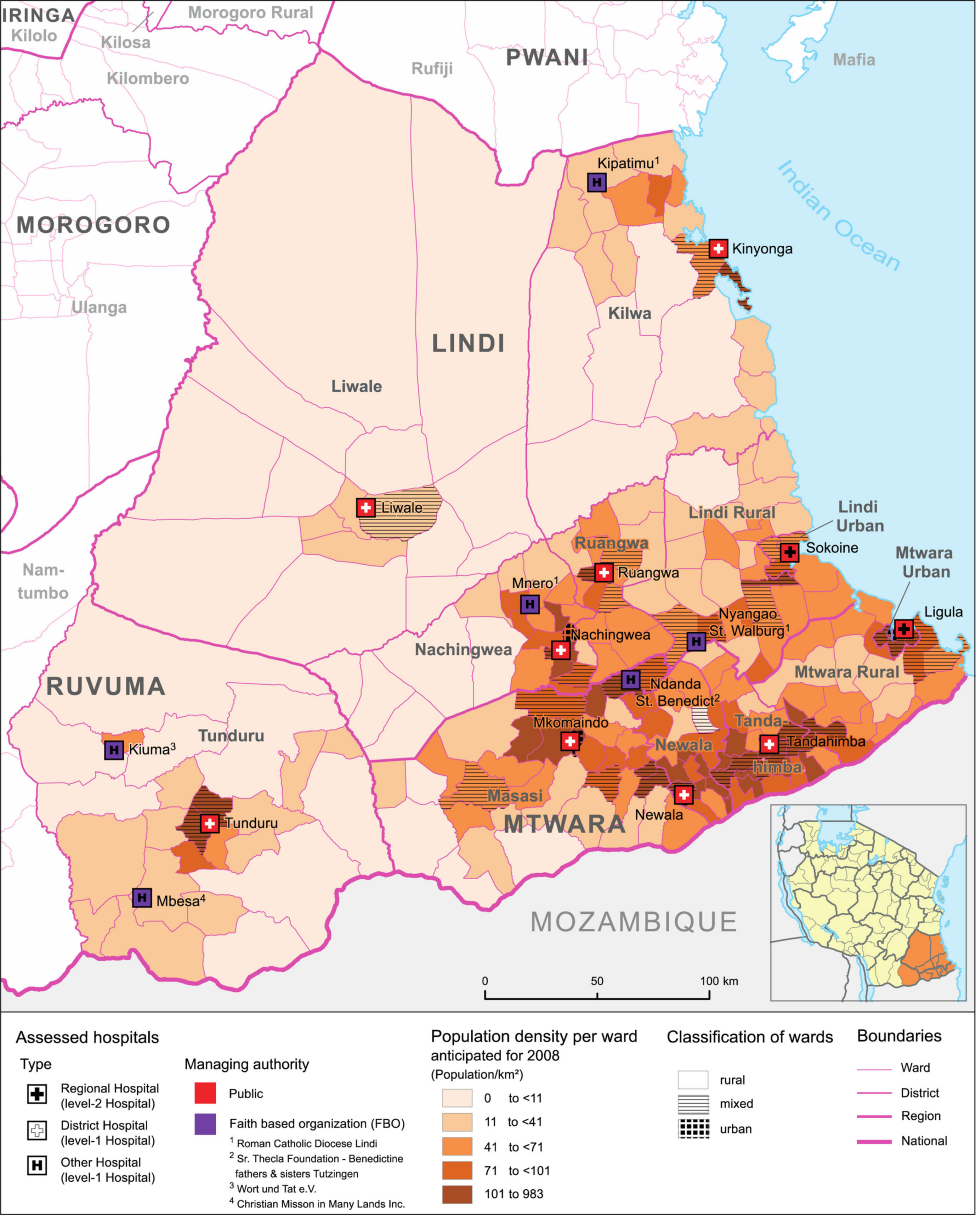
Hospital facilities in the study area by managing authority. Population densities by ward reflect estimates for 2008 and are based on the 2002 Population and Housing Census (24), adjusted for annual population growth (25). Ward classification was likewise derived from the Census. Layout by Beuth University of Applied Sciences (Berlin).

A total of 407 health facilities were reported by district authorities (dispensaries *n*=354; health centres *n*=37; hospitals *n*=16), which translates into an average facility density of 1.6 facilities per 10,000 people. The public sector is the primary health care provider and runs 87.5% (*n*=356/407) of health facilities (89.3% of dispensaries; 83.8% of health centres; 62.5% of hospitals). Among private providers, FBOs are the main contributors to health facility infrastructure, accounting for 5.1% of dispensaries, 10.8% of health centres and 37.5% of hospitals. All hospitals in the study area are under public (*n*=10) or FBO (*n*=6) management. FBO hospital location was classified as either rural (*n*=4) or mixed (*n*=2), whereas all public hospitals operate in either urban (*n*=4) or mixed (*n*=6) locations (Fig. 1).

### Hospital maternal health service capacity

#### Human resources

Only two obstetricians/gynaecologists were employed between all 16 hospitals (Table 2), reflecting a strong reliance on mid-level cadres. Both were assigned at the Regional Hospital in Lindi, with one of them being an expatriate working in a short-term contractual arrangement.No hospital had the complete set of specialists required. The majority of MDs employed in FBO hospitals had a further specialization (69.2%; *n*=9/13), which differed from the situation in public hospitals (39.1%; *n*=9/23). The six FBO hospitals also employed a higher number of expatriate MDs, compared to their 10 public counterparts (*n*=5 vs. *n*=3, respectively). Nurses were the main cadre providing qualified maternal health specific services. Although 70.6% (*n*=96/136) of nurses were employed in public hospitals, FBOs employed more than half (53.7%; *n*=22/41) of the more qualified registered nurses, which is also reflected by the composition of their nurse workforce: 55.0% of FBO nurses held the qualification of a registered nurse in contrast to 19.8% of public nurses.

**Table 2:** Selected hospital maternal health service indicators by managing authority

	FBO hospitals (*n*=6)		Public hospitals (*n*=10)		Total
		
	*n*	%	*n*	%	*n*
Maternal health specific personnel					
Obstetricians/Gynaecologists	0	0.0	2	100	2
Maternity nurses	40	29.4	96	70.6	136
Registered nurses	22	53.7	19	46.3	41
Enrolled nurses	18	18.9	77	81.1	95
Other related & skilled personnel					
Medical doctors (total)	13	36.1	23	63.9	36
Assistant medical officers	20	26.3	56	73.7	76
Clinical officers	25	19.8	101	80.2	126
Maternity and delivery beds					
Maternity beds	184	37.2	311	62.8	495
Delivery beds	23	35.4	42	64.6	65
Patient turnover (01–12/2008)					
Antenatal care	3,126	25.5	9,148	74.5	12,274
Births total	4,014	20.9	15,208	79.1	19,222
Normal deliveries	3,121	18.8	13,443	81.2	16,564
C-sections	893	33.6	1,765	66.4	2,658

Data derived from hospital survey. FBO = faith-based organization.

We detected substantial inconsistencies between the number of FBO employees reported by district authorities and the situation assessed on the ground. In the 10 districts in which the district questionnaire was applied, FBO hospitals alone employed more MDs, AMOs, COs and nurses than districts reported for all FBO health facilities in the region combined. When comparing the district to the FBO hospital data, the number MDs increased 2.6-fold (from *n*=5 to *n*=13), that of AMOs 6.7-fold (from *n*=3 to *n*=20), that of COs 1.7-fold (from *n*=15 to *n*=25) and that of nurses 2.5-fold (from *n*=70 to *n*=177). The staffing levels recorded in public hospitals matched the figures reported by the district respondents.

#### Patient turnover and official provider-fees

A patient turnover of 31,496 patients was recorded for ANC, normal deliveries and c-sections combined (period reviewed: January–December 2008). FBO hospitals accounted for 25.5% (*n*=3,126/12,274) of recorded ANC patient visits, 18.8% (*n*=3,122/16,564) of normal deliveries and 33.6% (*n*=893/2,658) of c-sections. The overall proportion of hospital births delivered by c-section was 13.8% (*n*=2,658/19,222). Pregnant women were reportedly exempted from official provider-fees for obstetric services in all public hospitals, whereas only two out of six FBO hospitals offered this exemption (Kipatimu Hospital in Kilwa District, which signed a Service Agreement in 2008 and Kiuma Hospital in Tunduru District). The remaining four FBO hospitals charged 4,000 TZS/3.33 USD (range 1,000–6,000 TZS/0.83–5.0 USD) on average for normal deliveries, and 12,000 TZS/10 USD (range 10,000–16,000 TZS/8.33–13.32 USD) for c-sections.

#### Capacity for maternal health services

Average service capacity was significantly higher in FBO hospitals compared to their public counterparts for all but one of the selected indicators (Table 3). Relative to the number of hospital births, FBO hospitals had a higher number of maternity nurses, AMOs and MDs, as well as maternity and delivery beds at their disposal. Further, the proportion of births delivered by c-section was twice as high compared to public hospitals.

**Table 3:** Hospital maternal health service capacity by managing authority

Maternal health indicators	FBO (*n*=6) Average of rates	Public (*n*=10) Average of rates	Average difference (95% CI)	*p* Value
Maternity & delivery beds				
Maternity beds per 1,000 births	47.19	20.65	26.54 (12.91, 40.17)	<0.001
Delivery beds per 1,000 normal deliveries	9.72	3.32	6.40 (3.05, 9.75)	0.001
Human resources				
Maternity nurses per 1,000 births	12.08	6.48	5.61 (1.72, 9.50)	0.008
Clinical officers per 1,000 births	8.41	7.28	1.13 (−3.61, 5.87)	0.618
MDs & AMOs per 1,000 births	8.14	5.09	3.05 (1.15, 4.96)	0.004
Proportion of c-sections				
C-sections per 100 births	22.72	11.48	11.24 (1.90, 20.59)	0.02

Data derived from hospital survey. Patient turnover (normal deliveries and c-sections) as recorded for the period January–December 2008. Student's *t*-test was applied. 95% CI=95% confidence interval; FBO=faith-based organization; MDs=medical doctors; AMOs=assistant medical officers.

### Patient flows and socio-geographic characteristics of service recipients

We performed descriptive spatial analysis to map the distribution of hospital births, patient flows and socio-geographic characteristics of hospital maternal health service recipients.

#### Linking hospital with national census data

We assigned 86.1% (*n*=27,124/31,496) of recorded patient visits to their ward of origin. In 4,372 cases, origin either could not be assigned to the existing NBS dataset (*n*=3,331) or had to be dropped due to village/town name duplicates (*n*=1,041). The majority of patient visits were traced to mixed (46.2%) or rural (42.3%) wards, while only 11.5% originated from urban areas.

#### Distribution of hospitals births in the study area

**Fig. 2:**
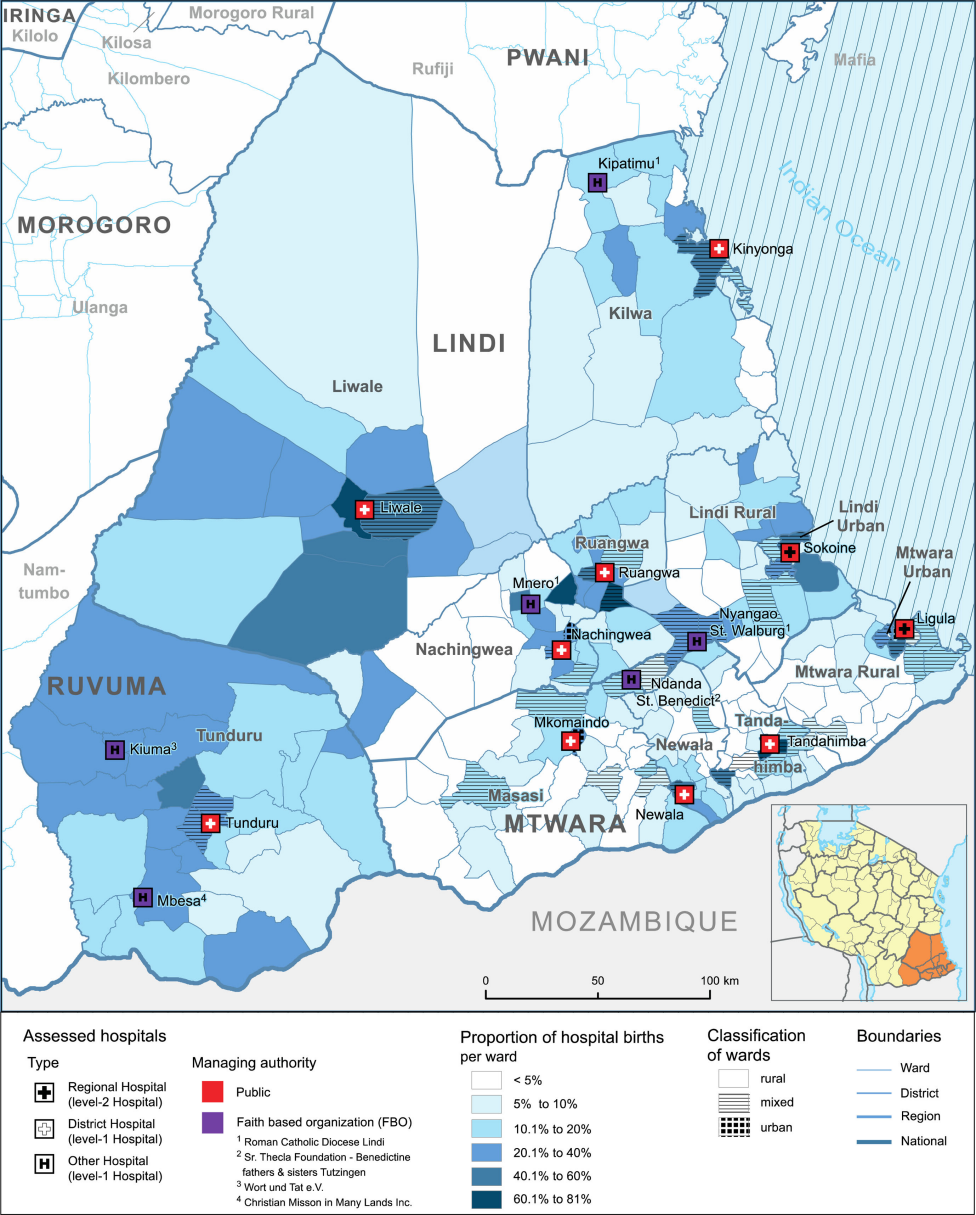
Proportion of hospital births by ward. Proportion of hospital deliveries by ward is based on the number of hospital births (numerator) and the anticipated number of births (denominator). Anticipated number of births were calculated on the basis of the 2002 Population and Housing Census, adjusted for annual population growth (25) and a crude birth rate of 38,1 per 10,000 population. Ward classification was likewise derived from the Census. Layout by Beuth University of Applied Sciences (Berlin).

The number of births in the study area was approximated to 96,805 for the period reviewed. Compared to the overall number of recorded hospital births (*n*=19,222), this would correspond to 19.9% of women, who chose to deliver in a hospital level facility and an estimated 2.7% who delivered by c-section. Figure 2 shows the proportion of hospital births by ward (please see Supplementary file for a mapping of the underlying patient flows). Overall, percentages of hospital births varied between different wards, districts and regions. The percentage of hospital births was approximately 11.2% in Mtwara Region, 15.1% in Lindi Region, and 18.9% in Tunduru District of Ruvuma Region. It was twice as high in non-rural (urban and mixed) compared to rural wards (25.1% vs. 11.4%, Chi-square test: *p*<0.001). Urban and mixed wards are primarily located in close proximity to hospitals, especially to those managed by public authorities.

#### Patient flows

Public district and FBO level-1 hospitals formally represent referral facilities for the districts in which they are located, whereas regional hospitals (level-2) serve both, their districts and their respective regions. A mapping of patient flows between hospitals and wards is provided in Supplementary file. The majority of 79.6% (*n*=11,291/14,190) of hospital patients residing in districts with hospital infrastructure also delivered their children in their home district. This means that they adhered to the formal catchment area of these hospitals. This figure excludes patients from Mtwara Rural, which does not dispose of a level-1 hospital of its own, but is served by the neighbouring regional hospital in Mtwara Urban. Excluding the two regional hospitals, FBOs recruited 27.7% (*n*=740/2,672) of patients who had a normal delivery from neighbouring districts in contrast to a share of 14.7% (*n*=1,165/7,917) in public district hospitals. This is largely caused by FBO managed Nyangao and Ndanda hospitals, which are both located close to their respective district borders. In Ndanda, 49.3% (*n*=431/874) of normal deliveries originated from outside Masasi District, whereas in Nyangao Hospital, 44.9% (*n*=79/176) of c-sections originated from outside Lindi Rural. Further, the actual catchment areas of public and FBO hospitals often overlap, particularly in the southern districts of Lindi Region, the northern parts of Mtwara Region, and in the eastern parts of Tunduru District of Ruvuma Region, where the linear distance between public and FBO hospitals seldom exceeds 50 km. In the four districts with co-existing public and FBO hospital infrastructure (Kilwa, Nachingwea, Masasi and Tunduru), 29.7% (*n*=1,654/5,561) of patients chose to deliver in FBO hospitals (Table 4). In these districts, FBOs accounted for 27.6% (*n*=1,318/4,777) of normal deliveries and 47.9% (*n*=316/660) of c-sections (please also see Supplementary file).

**Table 4:** Provider choice in districts with co-existing public and FBO hospital infrastructure

	Patient origin	Service performed in patients’ home district		
	
Service	(district)	*n* (%)^a^	Public *n* (%)^b^	FBO *n* (%)^b^
Normal deliveries	Kilwa	956 (90.6)	774 (81.0)	182 (19.0)
	Nachingwea	758 (70.2)	560 (73.9)	198 (26.1)
	Masasi	1,248 (85.5)	805 (64.5)	443 (35.5)
	Tunduru	1,815 (92.2)	1,320 (72.7)	495 (27.3)
	Total	4,777 (85.9)	3,459 (72.4)	1,318 (27.6)
				
C-sections	Kilwa	116 (86.6)	103 (88.8)	13 (11.2)
	Nachingwea	85 (75.9)	50 (58.8)	35 (41.2)
	Masasi^c^	–	–	–
	Tunduru	459 (97.0)	191 (41.6)	268 (58.4)
	Total	660 (91.8)	344 (52.1)	316 (47.9)

Patient turnover as recorded for the period January–December 2008. FBO=faith-based organization.

a Percentage of traced patients delivering in their home district.

b Percentage of traced patients delivering in their home district in either public or FBO hospitals.

c Results not displayed due to insufficient tracing success (<70% of recorded c-sections) at the FBO hospital.

#### Socio-geographic characteristics of public and FBO maternal health service recipients

In accord with the primarily rural location of FBO hospitals, the share of women with a rural origin was higher among FBO service recipients. We quantified the level of emphasis FBOs place on serving rural patients and compared it to the patient composition in public hospitals. Percentage of rural patients among FBO vs. public health service recipients: ANC 53.4% (*n*=1,546/2,895) vs. 20.7% (*n*=1,816/8,782), Chi-square test *p*<0.001; normal deliveries 61.5% (*n*=1,643/2,672) vs. 48.8% (*n*=5,287/10,838), Chi-square test: *p*<0.001; c-sections 77.8% (*n*=418/537) vs. 55.4% (*n*=759/1,371), Chi-square test: *p*<0.001. Even after excluding the four public hospitals located in urban wards, this trend is sustained [ANC 53.4% (*n*=1,546/2,895) vs. 10.2% (*n*=553/5,420), Chi-square test: *p*<0.001;normal deliveries 61.5% (*n*=1,643/2,672) vs. 53.3% (*n*=3,381/6,338), Chi-square test: *p*<0.001; c-sections 77.8% (*n*=418/537) vs. 58.2% (*n*=505/868), Chi-square test: *p*<0.001].

## Discussion

Our study gives an insight into the capacities of public and FBO hospitals to provide maternal health care in southern Tanzania, as well as the population reached with these services. To the best of our knowledge, a combined mapping of service capacity, patient flows and socio-geographic characteristics of public and FBO maternal health service recipients has not been previously realized in Tanzania and its feasibility represents an important finding of this study.

### Hospital births and patient flows

According to Tanzania's district policies, normal deliveries can be performed at local dispensaries or health centres, whereas complicated cases requiring more sophisticated care should be referred to hospitals. Previous population-based research in southern Tanzania demonstrated high rates of primary health facility bypassing in favour of higher level hospital care (27–29). A community-based survey in five out of our 12 selected districts demonstrated that only 41% of births were performed in a health facility, with hospitals accounting for three times as many facility births as dispensaries and health centres combined (28). We estimated that 19.9% of anticipated births in the study area were delivered in hospitals, which would correspond to every second facility birth overall. This indicates that primary health facilities in the study area may not be used to their desired extent. We estimated that 2.7% of births in the study area were delivered by c-section, which is in line with previous findings describing rates of 3% (22). Although generalization is difficult, this underscores the recommended minimum proportion of c-sections at a population level of 5–10% (30).

The average percentage of hospital births was twice as high in non-rural (urban and mixed) compared to rural wards. This can be explained by the distribution of urban and mixed wards, which are primarily located in close proximity to hospitals, especially to those managed by public authorities. In addition, we mapped the actual catchment areas of public and FBO hospitals on the basis of patient flows and showed where they overlap. In these areas, cooperation between public and FBO hospitals could be particularly promising, as both serve the same population. This could include an improved coordination of outreach services as well as a more efficient and systematic referral of patients.

### Characteristics of FBO hospital maternal health services

FBOs in Tanzania have a good reputation and are commonly believed to provide better health services than public healthcare providers (31–34). We found that, relative to the respective number of patients, FBO hospitals had significantly higher numbers of core medical professionals, as well as maternity and delivery beds at their disposal. The finding differs from the common perception of a more severe human resource crisis in the private health sector in Tanzania (14). Yet, the shortage of health workers is calculated on the basis of staffing level guidelines and does not capture human resource availability relative to patient turnover. FBO hospitals also employed a greater number of more qualified registered nurses and the same number of medical specialists as the more numerous public hospitals. Since no hospital in the study area had the complete set of medical specialists required, FBOs’ pool of specialists could be utilized for a more efficient referral of patients in the region. This would require reliable information about the human resource situation in FBO facilities, which was not available at the district level. The data inconsistencies were most likely caused by deficits of the health management information system and incomplete reporting to the district level. The current system should be reevaluated, as it is the district data, which is reported to the regional and national level. The dimension by which FBO staffing levels were underestimated by public authorities could also imply that the shortfalls in FBO facilities may be more similar to the public sector than generally anticipated. Further, the average percentage of c-sections among women delivering in FBO hospitals was twice the percentage recorded in public hospitals. We did not evaluate clinical presentations in women undergoing c-sections, but further research is recommended to investigate reasons for the observed difference.

FBOs in Tanzania primarily operate in rural areas where they have a long-standing tradition as health service providers (8, 16). Yet, the level of emphasis FBOs place on serving rural populations has not been previously quantified, nor has it been compared to the situaion in public hospitals. We demonstrated that around 60–70% of women delivering in FBO hospitals originated from rural wards. These women are likely to be most affected by the official provider-fees for obstetric services charged in most FBO hospitals. Provider-fees were shown to be associated with a decrease of deliveries performed in medical facilities (35, 36), whereas increased government financing had the opposite effect (37). According to the 2007 Household Budget Survey (38), the average monthly income in rural households of Tanzania was 28,000 TSZ/23.31 USD. Compared to the average official provider-fees for c-sections charged in FBO hospitals (12,000 TSZ/10 USD), this means that rural patients had to spend approximately 40% of their monthly income to receive such services. In this context, the low social and economic status of women and their limited autonomy over their own health care and the household budget should not be overlooked (39). The 2007 Household Budget Survey stated that ‘high costs were the most frequent complaint about missionary [FBO] hospitals’ among patients, which was less of a concern in public health facilities (38). Accordingly, Tanzania's 2010 Demographic Health Survey found that women reported lacking money as the major barrier to access health services, while distance to the health facility had less influence (10). Even though the government of Tanzania is committed to universal provision of maternal health services free of charge, Kruk et al. (2008) demonstrated that 30% of women delivering in public hospitals paid ‘unofficial’ provider-fees. Still, these were less than 10% of the official provider-fees charged in FBO hospitals, where the overall costs for deliveries were more than twice as high compared to public hospitals (13).

### Limitations

Our study had several limitations. The National Census data used to calculate population size and anticipated number of births in the study area was collected in 2002 and was adjusted for annual population growth. This reflects an approximation and may be subject to bias. Further, it cannot be determined to which degree admission and delivery books were complete and accurate. We could not evaluate exact distances between villages/towns and hospitals, because GPS coordinates of the settlements in the study area were not available. Official provider-fees were recorded as reported by hospital respondents and therefore did not cover any possible unofficial fees.

## Conclusion

As the government of Tanzania and members of the donor community continue to engage with FBOs in health service delivery, it will become increasingly important to understand what these organizations have to offer, how they offer it and to whom. We conclude that the substantial contribution of FBOs to maternal health services and capacity makes them valuable partners in health. The dependency of FBOs on official provider-fees for obstetric services and their significant emphasis on serving rural populations favours the extension of public provider-fee exemption policies to include FBO facilities. This could be achieved by a more widespread implementation of existing subsidiary contracts between private health facilities and local government authorities. Inclusive partnerships could increase integration of FBOs into the public health care system and improve coordination and the efficient use of resources. However, this relies on a steady and accurate flow of information from the private to the public health sector, which was found to be inadequate in the study area. We believe that improved recognition of the private health sector will also require better representation of FBOs in health system planning as well as continued advocacy for the partnership approach at all levels.

## References

[1] World Health Organization (WHO) (2013). Global Health Observatory (GHO). http://www.who.int/gho/maternal_health/mortality/maternal/en/.

[2] Nyamtema AS, Urassa DP, Massawe S, Massawe A, Lindmark G, van Roosmalen J (2008). Staffing needs for quality perinatal care in Tanzania. Afr J Reprod Health.

[3] Campbell OM, Graham WJ (2006). Strategies for reducing maternal mortality: getting on with what works. Lancet.

[4] Adegoke AA, van den Broek N (2009). Skilled birth attendance-lessons learnt. Int J Gynaecol Obstet.

[5] Yadamsuren B, Merialdi M, Davaadorj I, Requejo JH, Betran AP, Ahmad A (2010). Tracking maternal mortality declines in Mongolia between 1992 and 2007: the importance of collaboration. Bull World Health Organ.

[6] Nishtar S (2004). Public – private ‘partnerships’ in health – a global call to action. Health Res Policy Syst.

[7] World Health Organization (WHO) (2007). Faith-Based Organizations play a major role in HIV/AIDS care and treatment in sub-Saharan Africa. http://www.who.int/mediacentre/news/notes/2007/np05/en/index.html.

[8] Widmer M, Betran AP, Merialdi M, Requejo J, Karpf T (2011). The role of faith-based organizations in maternal and newborn health care in Africa. Int J Gynaecol Obstet.

[9] Vogel JP, Betran AP, Widmer M, Souza JP, Gulmezoglu AM, Seuc A (2012). Role of faith-based and nongovernment organizations in the provision of obstetric services in 3 African countries. Am J Obstet Gynecol.

[10] Tanzania National Bureau of Statistics (NBS), ICF Macro (2011). Tanzania demographic health survey 2010. http://www.measuredhs.com/pubs/pdf/FR243/FR243[24June2011].pdf.

[11] Tanzania Ministry of Health (MoH) (2003). National health policy. http://www.moh.go.tz/documents/nhp_finaldraft_24.10.03.pdf.

[12] Winani K (2011). Maternal and newborn health in Tanzania. Int J Gynaecol Obstet.

[13] Kruk ME, Mbaruku G, Rockers PC, Galea S (2008). User fee exemptions are not enough: out-of-pocket payments for ‘free’ delivery services in rural Tanzania. Trop Med Int Health.

[14] Tanzania Ministry of Health and Social Welfare (MoHSW) (2008). Annual health statistical abstract – Tanzania mainland 2008. http://www.moh.go.tz/documents/HMIS%20Abstract%202008.pdf.

[15] Tanzania Ministry of Health and Social Welfare (MoHSW) (2008). The national road map strategic plan to accelerate reduction of maternal, newborn and child death in Tanzania 2008–2015. http://www.unfpa.org/sowmy/resources/docs/library/R224_MOHTanzania_2008_Roadmap_MNCH.pdf.

[16] Tanzania Ministry of Health and Social Welfare (MoHSW) (2007). Primary Health Services Development Programme (PHSDP) 2007–2017. http://www.unfpa.org/sowmy/resources/docs/library/R222_MOHTanzania_2007_PHC_2007–2017.pdf.

[17] Tanzania Ministry of Health and Social Welfare (MoHSW) (2008). Health sector strategic plan III – partership for delivering the MDGs July 2009–June 2015. http://tanzania.um.dk/en/~/media/Tanzania/Documents/Health/Health%20Sector%20Strategic%20Plan%20III%20FINAL%20090408.pdf.

[18] Itika J, Mashindano O, Kessy F (2011). Success and constraints for improving public private partnership in health service delivery in Tanzania. ESFR Discussion Paper.

[19] Health Research for Action (HERA) (2005). Technical review 2005 – public private partnership for equitable provision of quality heath services. http://ihi.eprints.org/625/1/ihi.eprints.pdf_(63).pdf.

[20] Tanzania Nurses and Midwives Council (TNMC) (2007). Standard of profenciency for nursing education & practice in Tanzania. http://www.tnmc.go.tz/index.php?option=com_jdownloads&Itemid=85&view=finish&cid=19&catid=7&m=0.

[21] Songstad NG, Rekdal OB, Massay DA, Blystad A (2011). Perceived unfairness in working conditions: the case of public health services in Tanzania. BMC Health Serv Res.

[22] Nyamtema AS, Pemba SK, Mbaruku G, Rutasha FD, van RJ (2011). Tanzanian lessons in using non-physician clinicians to scale up comprehensive emergency obstetric care in remote and rural areas. Hum Resour Health.

[23] Tanzania Ministry of Finance and Economic Affairs (2010). Bank of Tanzania – annual report 2008/09. http://www.bot-tz.org/Publications/EconomicAndOperationsAnnualReports/June_2009.pdf.

[24] Tanzania National Bureau of Statistics (NBS) (2002). Tanzania national population and housing census 2002.

[25] The World Bank (2011). The World Bank Indicators. http://data.worldbank.org/indicator/SP.POP.GROW?page=1.

[26] United Nations High Commissioner for Refugees (1999). Reproductive health in refugee situations an inter-agency field manual. http://www.iawg.net/resources/iawg_Field%20Manual_1999.pdf.

[27] Kruk ME, Mbaruku G, McCord CW, Moran M, Rockers PC, Galea S (2009). Bypassing primary care facilities for childbirth: a population-based study in rural Tanzania. Health Policy Plan.

[28] Penfold S, Hill Z, Mrisho M, Manzi F, Tanner M, Mshinda H (2010). A large cross-sectional community-based study of newborn care practices in southern Tanzania. PLoS One.

[29] Kahabuka C, Kvale G, Moland KM, Hinderaker SG (2011). Why caretakers bypass Primary Health Care facilities for child care – a case from rural Tanzania. BMC Health Serv Res.

[30] Gibbons L, Belizán J, Lauer J, Betrán A, Merialdi M, Althabe F (2010). The global numbers and costs of additionally needed and unnecessary caesarean sections performed per year: overuse as a barrier to universal coverage – World Health Report (2010). Background Paper, No 30.

[31] Hutchinson PL, Do M, Agha S (2011). Measuring client satisfaction and the quality of family planning services: a comparative analysis of public and private health facilities in Tanzania, Kenya and Ghana. BMC Health Serv Res.

[32] Gilson L, Sen PD, Mohammed S, Mujinja P (1994). The potential of health sector non-governmental organizations: policy options. Health Policy Plan.

[33] Mamdani M, Bangser M (2004). Poor people's experiences of health services in Tanzania: a literature review. Reprod Health Matters.

[34] Pereira C, Mbaruku G, Nzabuhakwa C, Bergstrom S, McCord C (2011). Emergency obstetric surgery by non-physician clinicians in Tanzania. Int J Gynaecol Obstet.

[35] Stekelenburg J, Kyanamina S, Mukelabai M, Wolffers I, van Roosmalen J (2004). Waiting too long: low use of maternal health services in Kalabo, Zambia. Trop Med Int Health.

[36] Hotchkiss DR, Krasovec K, El-Idrissi MD, Eckert E, Karim AM (2005). The role of user charges and structural attributes of quality on the use of maternal health services in Morocco. Int J Health Plann Manage.

[37] Kruk ME, Galea S, Prescott M, Freedman LP (2007). Health care financing and utilization of maternal health services in developing countries. Health Policy Plan.

[38] Tanzania National Bureau of Statistics (NBS) (2009). Household budget survey 2007. http://www.nbs.go.tz/tnada/index.php/ddibrowser/2/datacollection.

[39] United Nations Children's Fund (UNICEF) (2011). Maternal and child health – UNICEF Tanzania. http://www.unicef.org/tanzania/6906_10741.html.

